# Machine Learning Applications Beyond Outcome Prediction in Plastic and Reconstructive Surgery: A Systematic Review of Diagnostic, Intraoperative, and Workflow Optimization Models

**DOI:** 10.7759/cureus.94825

**Published:** 2025-10-17

**Authors:** Abdulkreem Al-Juhani, Faisal Alzahrani, Omer Altamımı, Rodan Desoky, Lajeen Alnowaisser, Ibrahim Altamımı, Abdullah Esmail

**Affiliations:** 1 Forensic Medicine, Forensic Medicine Center, Jeddah, SAU; 2 Surgery, King Abdulaziz University Faculty of Medicine, Jeddah, SAU; 3 Otolaryngology - Head and Neck Surgery, King Saud University, Riyadh, SAU; 4 Surgery, King Abdulaziz University Hospital, Jeddah, SAU; 5 Medicine and Surgery, Alfaisal University College of Medicine, Riyadh, SAU; 6 Medicine, Al Maarefa University, Riyadh, SAU; 7 Surgery, Aldireyah Hospital, Riyadh, SAU; 8 Surgery, Dubai Academic Health Corporation, Dubai, ARE; 9 Clinical Sciences, Sulaiman Alrajhi University, Al Bukayriyah, SAU

**Keywords:** ai and machine learning, general plastic surgery, plastic and reconstructive surgery, plastic surgery, surgery

## Abstract

Plastic and reconstructive surgery increasingly depends on data-driven instruments to improve clinical decision-making. Machine learning (ML), through its capacity to analyze intricate, high-dimensional data, presents novel prospects for forecasting surgical outcomes with enhanced accuracy compared to traditional statistical models. This systematic study evaluates the extent, efficacy, and methodological rigor of ML applications in plastic and reconstructive surgery, encompassing burn treatment, microsurgical reconstruction, and breast surgery. Following the Preferred Reporting Items for Systematic Reviews and Meta-Analyses (PRISMA) 2020 standards, we searched four main databases for research published between January 2015 and March 2025. Eligible papers used ML models to predict clinical outcomes in plastic or reconstructive surgery and provided quantifiable performance indicators. Data were retrieved using the Checklist for Critical Appraisal and Data Extraction for Systematic Reviews of Prediction Modelling Studies (CHARMS) and Prediction Model Risk of Bias Assessment Tool (PROBAST) frameworks, then synthesized narratively. We included eleven studies that had more than 34,000 patients. Random forests, neural networks, and gradient boosting have emerged as the most prevalent and highest-performing models, with several attaining AUCs exceeding 0.90. Notwithstanding encouraging outcomes, the majority of research depended on internal validation, with only one performing external validation. Calibration reporting and data transparency were constrained. The majority of studies exhibited a high or moderate risk of bias. In summary, ML models provide significant prediction accuracy in plastic surgery; yet, they are limited by methodological deficiencies that impede clinical implementation. Future initiatives must prioritize external validation, repeatability, and ethical execution to facilitate the safe and successful incorporation into surgical practice.

## Introduction and background

Plastic and reconstructive surgery encompasses intricate procedures wherein precise outcome forecasting is crucial for patient safety and clinical strategizing. Conventional risk models frequently inadequately account for nonlinear interactions among various perioperative factors [1]. Machine learning (ML) has emerged as a viable option, adept at utilizing high-dimensional data to more accurately anticipate surgical results. In burn surgery, ML models have demonstrated significant efficacy in predicting death [1], classifying survival [2], and assessing postoperative infection risk [3]. Techniques such as random forests (RF), artificial neural networks (ANN), and gradient boosting have consistently exhibited superior accuracy compared to logistic regression (LR) in extensive datasets [1-3].

ML has been utilized for sepsis prediction in intensive care environments through automated learning frameworks, demonstrating exceptional discriminatory performance [4]. Likewise, prediction models for post-burn surgery mortality and sequelae have achieved area under the curve (AUC) values of 0.90 [5], while others have effectively identified patients at elevated risk of flap failure [6] or extended hospital stays [7]. In microsurgical reconstruction, ML approaches have been employed to predict postoperative problems and the likelihood of readmission. Models forecasting infection [3], multi-stage complications [8], and vascular compromise [9] have attained substantial accuracy and clinical interpretability.

Research utilizing national surgical databases has further illustrated the potential of ML in forecasting 30-day readmissions and necrosis after nipple-sparing mastectomy. Notwithstanding increasing interest, no previous synthesis has comprehensively assessed the application of ML in plastic and reconstructive surgery [9,10].

This review seeks to encapsulate contemporary ML applications for outcome prediction in this domain, contrasting model types, validation methodologies, and performance indicators, while emphasizing deficiencies in reporting and reproducibility.

Methodology

Protocol and Reporting Standards

This systematic review was performed in compliance with the Preferred Reporting Items for Systematic Reviews and Meta-Analyses (PRISMA 2020) standards. A comprehensive review protocol was established before the study to delineate the objectives, inclusion criteria, and analytical methodology, although it was not filed with PROSPERO. The objective was to synthesize and assess the application of ML models for forecasting clinical outcomes in plastic and reconstructive surgery, encompassing burns, microsurgical flaps, and breast reconstruction.

Eligibility Criteria

Studies that qualified adhered to the following criteria: (1) included human subjects undergoing plastic or reconstructive surgical procedures; (2) developed or validated ML models for predicting clinical or surgical outcomes; (3) reported quantitative performance metrics such as AUC, accuracy, sensitivity, or specificity; and (4) were published in English-language, peer-reviewed journals between January 2015 and March 2025. The included ML algorithms comprised both supervised methods (e.g., LR, RF, gradient boosting, neural networks, support vector machines (SVM)) and unsupervised techniques (e.g., k-means clustering). The primary outcomes of interest encompassed death, surgical complications, readmission, flap failure, vascular compromise, and surgical site infections. We excluded review papers, editorials, protocols, case reports, abstracts lacking full text, animal research, and studies that did not implement an ML framework.

Sources of Information and Search Methodology

A thorough literature review was conducted utilizing PubMed, Scopus, Web of Science, and IEEE Xplore to locate qualifying studies. The search concluded in March 2025 and encompassed publications published from January 2015 onwards. Search queries encompassed combinations of keywords and MeSH terms pertinent to ML and surgical disciplines, including (“machine learning” OR “artificial intelligence” OR “deep learning”) AND (“plastic surgery” OR “reconstructive surgery” OR “microsurgery” OR “burn surgery”) AND (“outcomes” OR “prediction” OR “mortality” OR “complications”). The inquiry was confined to publications in the English language. Furthermore, the reference lists of all included papers were meticulously examined to discover any papers not retrieved through database searches.

Data Extraction and Administration

A standardized data extraction form was created and pilot-tested utilizing the Checklist for Critical Appraisal and Data Extraction for Systematic Reviews of Prediction Modelling Studies (CHARMS) and Prediction Model Risk of Bias Assessment Tool (PROBAST) frameworks. For each study included, we extracted information on study metadata (author, year, country), population characteristics, surgical domain, sample size, ML algorithms employed, predicted outcomes, performance metrics, validation method (internal or external), feature selection strategy, management of missing data, model calibration, and availability of code or datasets.

Two reviewers conducted the data extraction independently, and any differences were addressed by discussion and re-evaluation of the original publication. Evaluation of the risk of bias for each included study was conducted using the PROBAST program. This instrument assesses predictive model research in four areas: participants, predictors, outcomes, and analysis. Each domain was assessed as exhibiting low, medium, or high bias risk. Two reviewers performed the evaluations independently, and discrepancies were reconciled through conversation.

The overall risk of bias for each study was assessed based on the highest domain-specific risk assessment, and the findings were compiled in a structured table. Due to the variability in study designs, ML algorithms, anticipated outcomes, and reporting standards, conducting a meta-analysis was impractical.

The findings were synthesized narratively, and the retrieved data were encapsulated in two structured tables: one detailing research characteristics and the other summarizing model parameters and prediction performance. No subgroup analysis or statistical aggregation of results was conducted.

## Review

Results

Process of Selecting Studies

**Figure 1:**
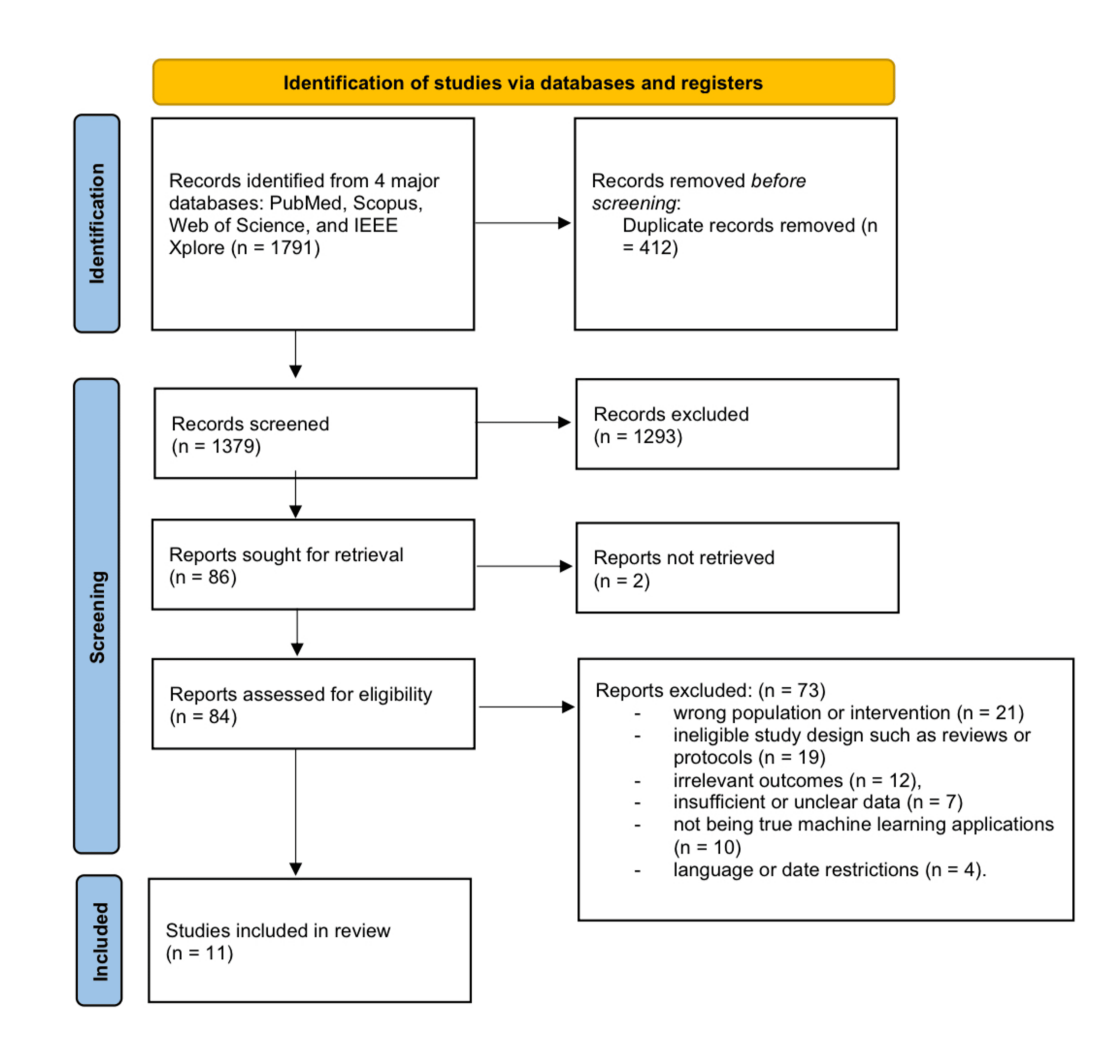
PRISMA flow diagram PRISMA: Preferred Reporting Items for Systematic Reviews and Meta-Analyses

Two reviewers separately evaluated the titles and abstracts of all obtained citations. Full-text publications were subsequently assessed for eligibility according to established inclusion and exclusion criteria. Disputes at any phase were settled through consensus or by seeking the opinion of a third reviewer. A PRISMA flow diagram was employed to record the study selection process, detailing the rationale for exclusion at each phase (Figure 1).

Study Identification and Overview

Eleven primary studies published from 2015 to 2025 fulfilled the qualifying criteria for this systematic review, encompassing approximately 34,000 patients and addressing three surgical domains: burn treatment (five studies), reconstructive microsurgery (three studies), and breast reconstruction (three studies). The geographic distribution comprised the United States (n = 6), Taiwan (n = 2), China (n = 1), the United Kingdom (n = 1), and South Korea (n = 1). Information is provided in Table 1. Sample sizes varied from approximately N = 211 (burn-ICU patients for sepsis prediction) to N ≈ 65,000 (national burn registry), with patient ages ranging from 18 to 85 years ([1-11]; Table 1).

**Table 1:** Summary of studies predicting postoperative or burn-related outcomes using clinical or registry data RCT: randomized controlled trial, NSQIP: National Surgical Quality Improvement Program, HCUP SID: Healthcare Cost and Utilization Project – State Inpatient Databases, NAC: nipple-areola complex, NSM: nipple-sparing mastectomy, DIEP: deep inferior epigastric perforator flap, ICU: intensive care unit, index hospitalization: initial hospital admission period during which surgery and immediate postoperative care occurred

Study (author, year)	Country	Study design	Target population	Sample size (N)	Outcome predicted	Event rate (%)	Data source	Follow‑up period
Stylianou et al., 2015 [1]	UK	Retrospective national registry	Adult burn patients	~65,000	In‑hospital mortality	~8	National burn injury database	During index hospitalization
Cobb et al., 2018 [2]	USA	Retrospective state inpatient analysis	Burn patients (state‑level dataset)	31,338	Survival (mortality vs. survival)	~6.4	HCUP state inpatient databases	Hospital stay
Kuo et al., 2018 [3]	Taiwan	Retrospective institutional cohort	Head and neck free‑flap patients	1,854	Surgical site infection (SSI)	Not stated	Single‑center surgical database	≤30 days postoperative
Tran et al., 2020 [4]	USA	Secondary analysis of multicenter RCT	Burn ICU patients	211	Sepsis prediction	Not stated	RCT/burn ICU registry	ICU admission
Park et al., 2022 [5]	South Korea	Retrospective burn ICU cohort	Critically burned surgical patients	731	90‑day postoperative mortality	Not stated	Burn ICU database	90 days after surgery
Shi et al., 2022 [6]	China	Multicenter retrospective cohort	Microvascular free‑flap reconstructions	946	Flap failure	3.6	Hospital operative records	Index hospitalization
Yeh et al., 2023 [7]	Taiwan	Retrospective single‑center study	High‑risk adult burn inpatients	224	Prolonged stay, grafting, complications	Not stated	Burn center electronic database	Index hospitalization
Kim et al., 2024 [8]	USA	Retrospective NSQIP registry analysis	Autologous breast reconstruction patients	14,274	Complication clustering (unsupervised)	14–30 (by cluster)	ACS NSQIP (2016–23)	30 days postoperative
Ozmen et al., 2025 [9]	USA	Retrospective NSQIP database analysis	DIEP flap breast reconstruction patients	13,312 †	30‑day hospital readmission	5.42 †	ACS NSQIP (2016–2022) †	30 days postoperative (readmissions) †
Meyer et al., 2025 [10]	South Korea	External validation using an institutional cohort	Nipple‑sparing mastectomy patients with implants	388	NAC necrosis	4.9	Institutional breast surgery registry	30 days postoperative
Yang et al., 2024 [11]	China	Single‑center retrospective cohort	Microvascular free‑flap surgery patients	570	Vascular complication	8.1	Hospital operative database	Index postoperative stay

Modeling Techniques and Validation Strategies

In the reviewed studies, various supervised ML algorithms were utilized, predominantly RF (n = 8), gradient boosting (including stochastic gradient boosting (SGB)/extreme gradient boosting (XGBoost)) (n = 6), ANN (n = 5), SVM (n = 4), LR (n = 3), and k-nearest neighbors (k-NN) (n = 2). A study utilized unsupervised clustering (k-means) to categorize patients based on risk. Internal validation was employed in all studies except one, generally using split-sample or k-fold cross-validation. Only Meyer et al. (2025) [10] conducted external validation, utilizing a previously trained RF model on an independent, non-overlapping cohort. Feature selection procedures and methods for addressing missing data were often inadequately recorded; however, one study (Kou et al. (2018) [3]) documented the elimination of missing cases and confirmed calibration based on the Brier score.

Predictive Performance

For models for burn care (mortality, survival, and sepsis), Stylianou et al. (2015) [1] devised and evaluated five models (LR, ANN, SVM, RF, and naïve Bayes (NB)) for forecasting in-hospital burn mortality in about 65,000 patients, with AUC values between 0.945 (RF) and 0.974 (ANN). The distinctions between LR (0.971) and ANN (0.974) were statistically insignificant. RF exhibited a marginally superior positive predictive value.

Cobb et al. (2018) [2] examined N = 31,338 inpatients to forecast survival; RF attained an AUC of 0.82, but SGB exhibited superior performance with an AUC of 0.93 [1,2]. Park et al. (2022) [5] assessed five ML models in 731 post-burn surgery patients; the RF model attained the highest AUC of 0.922 (95% CI: 0.902-0.942), substantially surpassing SVM, LR, and decision tree models (p < 0.001). Tran et al. (2020) [4] created an automated ML platform for 211 burn ICU patients; the optimal k-NN model achieved an AUC of 0.96, an accuracy of 89.7%, and a sensitivity of 95.8%, surpassing a logistic model (AUC 0.96) that utilized more features ([3,11]). Reconstructive microsurgery (surgical site infection, flap failure, vascular impairment).

Kuo et al. (2018) [4] developed ANN models to forecast surgical site infections following head and neck free-flap reconstruction; the postoperative ANN achieved an AUC of 0.892, compared to LR with a preoperative model AUC of 0.808 and a postoperative AUC of around 0.85, yielding a Brier score of approximately 0.09 [2,10].

Shi et al. (2022) [6] developed RF, SVM, and XGBoost classifiers for 946 microsurgical cases, reporting a flap failure rate of 3.6%. RF achieved the greatest AUC of 0.770, identifying age, BMI, and ischemia time as significant predictors. Yang et al. (2024) [11] conducted a comparative analysis of ANN, RF, and LR in forecasting postoperative vascular problems among 570 patients, with an event rate of 8.1%.

The ANN demonstrated superior discrimination (AUC 0.828; accuracy approximately 78%, sensitivity approximately 83%), surpassing RF (AUC approximately 0.80) and LR (AUC approximately 0.75) [7]. The model was applied to breast reconstruction outcomes (complication aggregation, readmission, and necrosis). Kim et al. (2024) [8] employed k-means clustering on 14,274 NSQIP cases to delineate seven subgroups categorized by complication rates (14-30%); no AUC metric applies to unsupervised clustering [8]. Ozmen et al. (2025) [9] examined 13,312 DIEP flap cases and created a stacked ML model to forecast 30-day readmission.

In the test set, the model demonstrated an accuracy of 88%, a recall of 79%, and an AUC of 0.8921 (95% CI: 0.853-0.927), with an event rate of 5.42%. Key predictors included early surgical site infection, operative duration, BMI, and preoperative albumin levels [1,2,9]. Meyer et al. (2025) [10] externally evaluated a trained RF model for predicting nipple-areola complex necrosis following nipple-sparing mastectomy, which had a 4.9% occurrence rate, using a cohort of 388 patients. The model attained an accuracy of 96% with moderate discrimination (AUC 0.70), demonstrating acceptable transportability despite a lower AUC [10]. The ML models tested and their performances are presented in Table 2.

**Table 2:** Model characteristics and performance AUC: area under the curve, ANN: artificial neural network, SVM: support vector machine, RF: random forest, NB: naïve Bayes, LR: logistic regression, SGB: stochastic gradient boosting, GBM: gradient boosting machine, XGBoost: extreme gradient boosting, LightGBM: light gradient boosting machine, DNN: deep neural network, k-NN: k-nearest neighbors, GAN: generative adversarial network, Acc: accuracy, Sens: sensitivity, Spec: specificity, CV: cross-validation, AutoML: automated machine learning

Study (year)	Outcomes (combined)	Models tested	Best‑performing model(s)	Notes (reported metrics and key findings)
Stylianou et al. (2015) [1]	Clinical outcome discrimination (study‑defined)	LR; ANN; SVM; RF; NB	No single best (no significant differences)	AUCs: ANN 0.974; LR 0.971; NB 0.970; SVM 0.967; RF 0.945. RF had the highest PPV but with lower sensitivity.
Cobb et al. (2018) [2]	Burn survival	RF; SGB (gradient boosting)	SGB	SGB showed superior discrimination for survival (AUC 0.93) vs. RF (AUC 0.82).
Kuo et al. (2018) [3]	Surgical outcome (preoperative; postoperative)	ANN; LR	ANN	Postoperative ANN AUC 0.892; preoperative ANN AUC 0.808; both ANN models significantly outperformed LR.
Tran et al. (2020) [4]	Sepsis prediction	LR; k‑NN; SVM; RF; GBM; NB; DNN (AutoML considered)	k‑NN (AutoML); LR (16‑feature traditional)	k‑NN (AutoML): AUC 0.96, Acc 89.7%, Sens 95.8. LR (16 features): AUC 0.96, Acc 86%. Several other models achieved AUC ≈ 0.92–0.95. Using only standard burn-sepsis criteria gave much lower performance (RF AUC ~ 0.76).
Park et al. (2022) [5]	Complication prediction	RF; AdaBoost; Decision Tree; SVM; LR	RF	RF AUC 0.922 (95% CI 0.902–0.942), Sens 66.2%, Spec 93.8. AdaBoost AUC ~ 0.90 (NS vs. RF, P = 0.36). SVM ~ 0.85 (worse than RF).
Shi et al. (2022) [6]	Failure prediction (study‑defined)	RF; SVM; XGBoost	RF	RF AUC 0.770 and ~ 96% accuracy (reflecting class imbalance, 3.6% positives); higher precision/recall than SVM (AUC ~ 0.68) and XGBoost (AUC ~ 0.74).
Yeh et al. (2023) [7]	Prolonged stay; skin graft needed; any complication	RF; XGBoost; LightGBM; LR	RF (tied with XGBoost for “any complication”)	Prolonged stay: RF 0.811; XGBoost 0.799; LightGBM 0.795. Skin graft needed: RF 0.788 (highest). Any complication: RF 0.872 and XGBoost 0.872 (tie).
Kim et al. (2024) [8]	Risk stratification via clustering	K‑means (unsupervised)	Unsupervised (no “best” predictive model)	Seven clusters identified with rising complication rates (~ 14% up to > 30%); no supervised AUC/accuracy metrics.
Ozmen et al. (2024) [9]	Methodological perspective (no model training)	GAN‑based synthetic data (perspective)	Not applicable (no predictive model)	Perspective article discussing synthetic data (GANs) to improve future model performance; no quantitative model metrics reported.
Meyer et al. (2025) [10]	NAC necrosis prediction (external validation)	RF (pre‑trained model)	RF (only model validated)	External validation: Acc 96%, AUC 0.70; Sens 74%, Spec 97%. Original training report noted ~ 97% accuracy.
Yang et al. (2024) [11]	Complication prediction	LR; RF; ANN	ANN	ANN AUC 0.828; Acc 78%, Sens 83%. RF ~ 0.80; LR ~ 0.75. ANN selected for deployment.

Risk of Bias Assessment

A systematic PROBAST evaluation (Table 3) identified a single study (Meyer et al. (2025) [10]) as possessing a low overall risk of bias, mainly attributable to external validation and sufficient calibration reporting. Five studies (Stylianou et al. (2015) [1], Kuo et al. (2018) [3], Shi et al. (2022) [6], Ozmen et al. (2025) [9], and Kim et al. (2024) [8]) were assessed as moderate risk, frequently attributable to absent calibration metrics or ambiguous methodologies for handling missing data. The five remaining studies (Cobb et al. (2018) [2], Tran et al. (2020) [4], Park et al. (2022) [5], Yeh et al. (2023) [7], and Yang et al. (2025) [11]) were assessed as high risk, primarily due to small sample sizes for the goal outcome, absence of external validation, and inadequate statistical transparency. Significantly, merely two investigations disclosed calibration performance (Brier score in Kuo et al. (2018) [3]; post-hoc calibration or Hosmer-Lemeshow in Meyer et al. (2025) [10]); none provided source code or datasets openly, hindering independent replication. The approaches for handling missing data and feature selection were often inadequately detailed, compromising reproducibility.

**Table 3:** Risk of bias assessment

Study (author, year)	Participants	Predictors	Outcome	Analysis	Overall risk
Stylianou et al. (2015) [1]	Low	Low	Low	Medium	Medium
Cobb et al. (2018) [2]	Low	Medium	Low	High	High
Kuo et al. (2018) [3]	Low	Medium	Low	Medium	Medium
Tran et al. (2020) [4]	Low	Low	Medium	High	High
Park et al. (2022) [5]	Medium	Medium	Medium	High	High
Shi et al. (2022) [6]	Low	Low	Low	Medium	Medium
Yeh et al. (2023) [7]	Low	Low	Low	High	High
Kim et al. (2024) [8]	Low	Low	Low	Medium	Medium
Ozmen et al. (2025) [9]	Low	Medium	Low	Medium	Medium
Meyer et al. (2025) [10]	Low	Low	Low	Low	Low
Yang et al. (2025) [11]	Medium	Medium	Low	High	High

Synthesis and Insight

The findings indicate the potential effectiveness of ML models, specifically ANN, RF, and gradient boosting techniques, in forecasting unfavorable surgical outcomes in cosmetic and reconstructive surgery. The majority of primary outcomes attain area under the curve values exceeding 0.85, and in certain instances, surpassing 0.95. The performance for predicting mortality and readmission was notably elevated in extensive datasets. Nonetheless, methodological constraints, including insufficient external validation, inadequate reporting of calibration or absent data, and restricted reproducibility, dampen enthusiasm for current clinical implementation.

Discussion

In plastic and reconstructive surgery, ML models demonstrate efficacy in the domains of burns, microsurgery, and breast reconstruction. This aligns with healthcare trends in which ML facilitates clinical decision-making [12-14]. Our findings indicate that RF and neural networks outperform conventional statistical approaches in terms of AUC, with some achieving values beyond 0.95 [1,2,5]. Extensive meta-analyses indicate that the superiority of ML over regression is constrained [13,15].

Nonetheless, ML models are becoming pertinent to clinical practice. In burn care, tools effectively predict sepsis, mortality, and survival with good calibration and discrimination [16]. ML has enabled microsurgeons to identify flap failure, vascular compromise, and surgical site infections at an early stage, hence enhancing preoperative planning and intraoperative monitoring [17]. ML-based risk categorization for complications in breast reconstruction facilitates customized surgical planning [9,10]. The application of ML in aesthetic surgery (forecasting cosmetic results and automating anatomical evaluations) demonstrates its adaptability and prevalence [18].

Nonetheless, methodological challenges persist. Most models employed internal validation, had restricted datasets, and lacked calibration measures or approaches for addressing missing data [19,20]. Certain deficiencies reduce generalizability and exaggerate actual performance in real-world scenarios. Certain investigations indicated inflated metrics lacking external evaluation, a concern prevalent among various surgical specialties [14,21]. Enhanced reporting and methodological rigor are required due to noncompliance with TRIPOD [20].

Reproducibility and transparency are constrained. The bulk of the examined studies did not disclose source code or datasets. AI research typically suffers from a deficiency in open science, hindering replication and advancement [22,23]. Transparency methodologies emphasize the interchange of code and data for external validation and enhancement of models [24]. In the absence of these methodologies, assertions regarding model performance are untrustworthy and erode physician confidence.

Another significant issue is interpretability. Surgeons must comprehend ML predictions. Explainable AI techniques such as SHAP and LIME can highlight feature contributions [25], while recent clinical studies question their usefulness [26]. Oversimplifying intricate model behavior or biases in training data may lead to false reassurance. Consequently, research must implement more robust, therapeutically effective interpretability frameworks.

Fairness and equity require thought. ML algorithms developed with biased datasets may sustain disparities in care [27]. Risk predictions for at-risk populations may lack precision due to the underrepresentation of minorities or gender disparities. Active auditing, prevention of algorithmic bias, and demographic transparency are essential. Equity must be evaluated throughout the model development process and thereafter. Failure to address these issues may lead to clinically unsafe disparities that disproportionately affect marginalized populations [28].

The accountability of decisions produced by ML is a significant ethical and legal concern. The liability for patient harm associated with models is somewhat ambiguous for clinicians, software developers, and institutions [29]. Clinical oversight and legal clarity necessitate well-defined governance systems, incorporating human-in-the-loop decision-making. Professional and legal standards for AI-assisted decision-making are endorsed. Researchers have proposed the establishment of specialized regulatory pathways for ML-based goods, as conventional approval processes may not adequately address their iterative characteristics [29,30].

Regulatory authorities and researchers react. DECIDE-AI [30], CONSORT-AI [31], and TRIPOD-AI establish standards for the creation and reporting of AI tools [20,32]. These standards direct the creation, reporting, and validation of AI tools prior to clinical application. Federated learning may address data sharing constraints and facilitate successful multicenter models while safeguarding patient privacy [33].

Clinicians are required to engage in ethical implementation. Surgeons require data science knowledge to effectively apply ML technologies [34]. Collaboration between clinicians and data scientists in model development guarantees relevance and practicality. The global guidelines established by WHO and the EU underscore the necessity for transparent, human-centered, and fair AI systems [35,36]. Surgical professional organizations have emphasized the necessity of surgeon oversight, transparency, and patient consent when using AI in surgical procedures [37-39].

Ultimately, a comprehensive real-world assessment is crucial for sustained success. External validation, multicenter prospective trials, and longitudinal studies must investigate the impact of ML on surgical outcomes, workflow, and patient safety [40,41]. Multidisciplinary AI oversight committees can evaluate new ML technologies for bias, safety, and relevance prior to their deployment in hospitals.

## Conclusions

Models like RF and neural networks sometimes surpass traditional methods in their predictive accuracy for surgical outcomes. This illustrates that ML possesses considerable potential in cosmetic and reconstructive surgery. Nonetheless, extensive clinical implementation is hindered by methodological deficiencies, including insufficient calibration reporting, inadequate external validation, and limited reproducibility. To guarantee that ML technologies are precise, interpretable, and equitable in practical surgical environments, future efforts must focus on stringent validation, transparent documentation, and ethical implementation.
